# Senataxin helicase, the causal gene defect in ALS4, is a significant modifier of C9orf72 ALS G4C2 and arginine-containing dipeptide repeat toxicity

**DOI:** 10.1186/s40478-023-01665-z

**Published:** 2023-10-17

**Authors:** Craig L. Bennett, Somasish Dastidar, Frederick J. Arnold, Spencer U. McKinstry, Cameron Stockford, Brian D. Freibaum, Bryce L. Sopher, Meilin Wu, Glen Seidner, William Joiner, J. Paul Taylor, Ryan J. H. West, Albert R. La Spada

**Affiliations:** 1 Departments of Pathology, Laboratory Medicine, Neurology, and Biological Chemistry, UCI Center for Neurotherapeutics, University of California Irvine School of Medicine, Irvine, CA 92697 USA; 2 Department of Neurology, Duke University School of Medicine, Durham, NC 27710 USA; 3 Center for Molecular Neurosciences, Kasturba Medical College, Manipal, 576104 India; 4 Department of Cell and Molecular Biology, St. Jude Children’s Research Hospital, Memphis, TN 38105 USA; 5 Department of Laboratory Medicine and Pathology, University of Washington Medical Center, Seattle, WA 98195 USA; 6 Department of Pharmacology, University of California, San Diego, La Jolla, CA 92093 USA; 7 Howard Hughes Medical Institute, Chevy Chase, MD 20815 USA; 8 Sheffield Institute for Translational Neuroscience, University of Sheffield, Sheffield, S10 2HQ UK; 9 Neuroscience Institute, University of Sheffield, Sheffield, S10 2TN UK; 10 Department of Neurobiology and Behavior, University of California Irvine School of Biosciences, Irvine, CA 92697 USA; 11 UCI Center for Neurotherapeutics, University of California Irvine, Irvine, CA 92697 USA

**Keywords:** Amyotrophic lateral sclerosis, C9orf72, Senataxin, Dipeptide repeat, Drosophila, Nucleolus

## Abstract

Identifying genetic modifiers of familial amyotrophic lateral sclerosis (ALS) may reveal targets for therapeutic modulation with potential application to sporadic ALS. GGGGCC (G4C2) repeat expansions in the *C9orf72* gene underlie the most common form of familial ALS, and generate toxic arginine-containing dipeptide repeats (DPRs), which interfere with membraneless organelles, such as the nucleolus. Here we considered senataxin (SETX), the genetic cause of ALS4, as a modifier of *C9orf72* ALS, because SETX is a nuclear helicase that may regulate RNA–protein interactions involved in ALS dysfunction. After documenting that decreased SETX expression enhances arginine-containing DPR toxicity and *C9orf72* repeat expansion toxicity in HEK293 cells and primary neurons, we generated SETX fly lines and evaluated the effect of SETX in flies expressing either (G4C2)_58_ repeats or glycine-arginine-50 [GR(50)] DPRs. We observed dramatic suppression of disease phenotypes in (G4C2)_58_ and GR(50) *Drosophila* models, and detected a striking relocalization of GR(50) out of the nucleolus in flies co-expressing SETX. Next-generation GR(1000) fly models, that show age-related motor deficits in climbing and movement assays, were similarly rescued with SETX co-expression. We noted that the physical interaction between SETX and arginine-containing DPRs is partially RNA-dependent. Finally, we directly assessed the nucleolus in cells expressing GR-DPRs, confirmed reduced mobility of proteins trafficking to the nucleolus upon GR-DPR expression, and found that SETX dosage modulated nucleolus liquidity in GR-DPR-expressing cells and motor neurons. These findings reveal a hitherto unknown connection between SETX function and cellular processes contributing to neuron demise in the most common form of familial ALS.

**Supplementary Information:**

The online version contains supplementary material available at 10.1186/s40478-023-01665-z.

## Introduction

Amyotrophic lateral sclerosis (ALS) is a heterogeneous group of progressive, adult-onset motor neuron disorders that typically lead to death from respiratory collapse 2–5 years after disease onset [35, 40]. While most cases of ALS appear to be sporadic without an obvious genetic cause or inheritance pattern, at least 10% of ALS cases are familial and result from a specific gene defect segregating within pedigrees as a Mendelian trait. Expansion of a GGGGCC (G4C2) repeat in an intron of the *C9orf72* gene has emerged as the most common cause of familial ALS as well as a frequent occurrence in sporadic ALS [29]. Pathogenic G4C2 repeat expansions at the *C9orf72* locus can number in the hundreds or thousands, while unaffected individuals usually carry *C9orf72* G4C2 alleles ranging from 2 to 23 repeats in length [17, 38]. Because *C9orf72* ALS is remarkably common, great effort has been placed on defining its mechanistic basis, and three leading models for disease pathogenesis have emerged: (1) RNA gain-of-function, (2) loss of *C9orf72* normal function, and (3) production of toxic dipeptide repeat (DPR) proteins via Repeat Associated Non-ATG (RAN) translation. By studying the genetic defects and histopathology underlying many different forms of familial ALS and comparing these findings to the molecular abnormalities and cell pathology present in sporadic ALS, independent lines of investigation have coalesced on dysregulation of RNA metabolism as a potential unifying feature of disease pathogenesis. Central to this work has been the realization that toxic arginine-containing DPRs, specifically poly glycine-arginine (GR) and poly proline-arginine (PR) produced by the *C9orf72* G4C2 repeat expansion, can interfere with a number of fundamental pathways in the cell, and that such arginine-containing DPRs have a proclivity to interact with and disrupt the function of low-complexity domain proteins involved in the formation of membraneless organelles, including the nucleolus [19, 25].

Amyotrophic lateral sclerosis type 4 (ALS4) is a juvenile-onset familial form of ALS characterized by dominant inheritance and slowly progressive motor neuronopathy [37]. ALS4 is exceedingly rare, with very few families ascertained worldwide, and in 2004, ALS4 was found to be caused by mutations in the senataxin gene [14]. Senataxin mutations in ALS4 are presumably gain-of-function, as loss-of-function mutations in the senataxin gene are responsible for autosomal recessive Ataxia with Oculomotor Apraxia type 2 (AOA2), and AOA2 carriers do not develop motor neuron disease [33], though this does not rule out a role for altered senataxin function in ALS4 pathogenesis, as occurs in dominant polyglutamine repeat expansion disease [26]. Senataxin (SETX) is a large 2677 amino acid protein that contains an amino-terminal, protein-interaction domain and a conserved, carboxy-terminal helicase domain which defines it as a superfamily I, RNA–DNA helicase [46].

Many different studies have shown that arginine-containing DPRs interact with proteins in the nucleolus to disrupt liquid–liquid phase separation of low complexity domain proteins critical to the function of this organelle (*reviewed in* [44]). With a shift towards greater emphasis on the role of longer DPRs (≥ 80-repeats) in ALS, GR repeats were shown to cause nucleolar stress in a length-dependent manner [32], and very long GFP-GR repeat expression constructs [e.g. GR(1136)] also showed distinct nucleolus localization in HeLa cells, as do shorter GR(36) repeats [8, 48]. Furthermore, nuclear exosome components are powerful modifiers of *C9orf72* G4C2 toxicity in *Drosophila* [18], and helicase proteins can modulate RNA–protein interactions to regulate the assembly and function of membraneless organelles [30].

We thus hypothesized that the SETX helicase might protect against *C9orf72* toxicity. To test this, we examined the effect of SETX on *C9orf72* toxicity in several in vitro and in vivo model systems. We found that reduced SETX expression enhanced *C9orf72* G4C2 repeat expansion toxicity and arginine-containing DPR toxicity in mammalian cells and primary neurons. We then documented that SETX expression fully rescued severe degenerative phenotypes and prevented lethality in crosses with the GR(50) *Drosophila* model. Furthermore, C9orf72 fly models expressing 1000 GR repeats that reveal age-related motor deficits were rescued with SETX co-expression. We found that physical interaction between SETX and arginine-containing DPRs is partially RNA-dependent, and when we performed fluorescence recovery after photobleaching (FRAP) on the nucleolus of HEK293 cells expressing Nucleolin-mCherry and GR(50), we noted that SETX significantly increased the kinetics of recovery of the mCherry signal in the nucleolus. This phenomenon was confirmed in induced pluripotent stem cell derived motor neurons from an ALS4 human patient and matched isogenic control lines. Our results indicate that SETX is a powerful modifier of arginine-containing DPR toxicity and can regulate the homeostasis of membraneless organelles whose assembly and function involves RNA-RNA and RNA–protein interactions.

## Materials and methods

### Expression constructs

The P-LoopΔ-SETX construct was generated using the Gibson Assembly Cloning Kit (NE BioLabs™) to create an eight amino acid deletion (1963–1970) of the SETX GTP/ATP binding domain (GPPGTGKS) as described recently [7]. A range of expression plasmids to study DPR toxicity were kindly provided by the laboratory of Zheng Ying, including GFP; GFP-GA_30_; GFP-GP_30_, GFP-GR_30_, GFP-PA_30_ and GFP-PR_30_ [45]. The GFP, GFP-GR_30_ and GFP-GA_30_ synthetic constructs were subcloned into the pSico lentiviral vector for efficient transduction studies, Sanger sequencing analysis was performed to confirm sequence integrity, and visual inspection of trial infections confirmed translation of GFP-fusion protein. Lentivirus particles were produced by triple transfecting pMD2.G, R8.74 and GFP-GR-30, GFP-GA_30_ or empty vector into HEK293 cells according to standard protocol. For siRNA knockdown experiments, HEK293 cells were transiently transfected for 48 h with a predesigned Silencer Select siRNA to SETX (s22951) or a Scramble control (si-CRL), using RNAiMAX™ Reagent (Invitrogen) at a final concentration of 10 nM.

### Cell culture studies

Primary cortical neurons (PCN) were cultured from dissociated cortex of postnatal day-0 to day-2 (P0-P2) C57BL/6 J pups as described previously [52]. Dissociation was performed using Trypsin (T9935), Trypsin Inhibitor (T6522), and DNase I (Roche). Primary neurons were seeded onto plates coated with (0.1 mg/mL) poly-D-lysine hydrobromide (P1024) and grown in complete media (CM) consisting of Neurobasal-A medium (Thermo Fisher Scientific) supplemented with 0.5 mM L-glutamine (Thermo Fisher Scientific), 0.25% penicillin–streptomycin (Thermo Fisher Scientific), and 0.25% B-27 supplement (Thermo Fisher Scientific). PCN were maintained until treatment by removing half of the CM and replacing it with fresh CM on day 3 (where day 0 is the day of cell seeding), day 5, and every second day after day 5.

To quantify cell death, we employed a Propidium Iodide (PI) exclusion assay and a lactate dehydrogenase (LDH) cell death assay. PI is a membrane impermeable stain and therefore does not enter viable cells with intact membranes. When PI does gain access to nucleic acids and intercalates, its fluorescence increases dramatically and is therefore used to identify dead cells. To perform this assay, we removed media from each sample being careful not to dislodge adherent cells and did not rinse with PBS. We then incubated in replacement media at 37 °C containing PI (1:3000) and Hoechst dye (1:10,000) for 15 min before imaging under a Zeiss 780 LSM confocal microscope at 10X objective magnification. Briefly 3 images were taken per well and each experiment was performed in at least triplicate. The lactate dehydrogenase (LDH) assay was conducted using LDH-Cytotoxicity Assay Kit II (BioVision, K313-500-2) according to the manufacturer’s instructions. Briefly, LDH Assay Buffer and WST Substrate Mix were added to media harvested from primary neurons. Plate readings were taken at four time points: 30, 60, 90, and 120 min. The percent of LDH released was calculated as the percent cytotoxicity normalized to the amount of total protein per well (μg/μL). Amount of protein was separately determined using BCA assay using the Pierce BCA Protein Assay Kit (Cat#23225). High control cells were treated with 10 μl of the cell lysis buffer provided with the LDH assay kit and kept in the incubator for 20 min before the media was harvested for LHD assay. For Low Control the cells/neurons were treated with complete media for neuronal growth.

### Western blot, immunoprecipitation and FRAP assay

#### Western blot analysis

Protein lysates from whole brain, spinal cord tissue or cell line extracts were prepared as previously described [43]. We loaded 30–50 µg of homogenized proteins per lane, and after running 3–8% Tris–Acetate gels (Invitrogen), samples were transferred to PVDF membranes (Millipore), which were blocked in 3% milk in PBS at RT for 1 h. Membranes were incubated with an anti-SETX Ab (A301-105A, Bethyl), anti-Flag Ab (F1804, Sigma), β-actin (ab8226, AbCam), and eGFP (A-11122, Invitrogen) in PBS-T with 3% BSA at 4 °C overnight. The primary antibody was visualized with horseradish-peroxidase conjugated anti-rabbit or anti-mouse IgG (Santa Cruz) at 1:5000 dilution and enhanced chemiluminescence (Amersham). Densitometry analysis was performed using the NIH ImageJ software application and normalized to β-actin signal intensity.

#### Immunoprecipitation

Cells were rinsed twice with ice-cold PBS and lysed in ice-cold lysis buffer (25 mM HEPES–KOH pH 7.4, 150 mM NaCl, 5 mM EDTA, 1% Triton X-10040 mM, one tablet of EDTA-free protease inhibitors (#11873580001 from Roche) per 10 mL of lysis buffer. The soluble fractions from cell lysates were isolated by centrifugation at 12,000 rpm for 11 min in a microfuge. Protein lysates were quantified using Pierce BCA Protein Assay Kit (Thermo Scientific) following the manufactures protocol. For immunoprecipitations, primary antibodies were incubated with Dynabeads® (Invitrogen) overnight, and then washed with sterile PBS. Antibodies bound to Dynabeads were then incubated with lysates with rotation for 2 h at 4 °C. Immunoprecipitates were washed three times with lysis buffer. Immunoprecipitated proteins were denatured by the addition of 20 µl of sample buffer and heated for 10 min at 70 °C, resolved by SDS-PAGE, and analyzed via Western blot analysis.

#### Fluorescence recovery after photobleaching (FRAP)

HEK293 cells were transfected with three constructs (BFP or BFP-GR_50_, GFP or SETX-GFP, mCherry-Nucleolin), and grown for 48 h in 1% FBS in DMEM to slow cell division. Cells were imaged on a Nikon Confocal (*A1 HD25/A1R HD25*). Cells were chosen with clear expression of all three fluorophores, distinct nucleoli, and expression of GR50 in the nucleolus if expressed. High levels of SETX are associated with diffuse Nucleolin, likely due to S-phase arrest [7]. Thus, such cells were excluded from analysis. Nucleoli were bleached and recovery was recorded every 2 s for 90 s. 40 cells per condition were imaged, and any cells whose focus shifted during the imaging time were discarded. FRAP was calculated by correcting for background measurements, normalizing to a region of the nucleoplasm, and setting the initial bleaching reading to zero. We did not bleach the entire nucleolus as FRAP rate does not appear to be dependent on the size of the bleached region. Bleaching a larger region (an entire large nucleolus ranging from 0.5 to 3 μm in diameter) reduces the level of fluorescence intensity at the maximum recovery. Results were fit to a curve using the One Phase Association function in Graphpad Prism. Plateau values were used to determine mobile fraction, and K and half-time values used to determine dynamics. These values were analyzed by one-way ANOVA and pairwise comparisons.

### Drosophila studies

#### UAS-Gal4 expression of wt-SETX and L389S-SETX in Drosophila

Using standard breeding strategy and maintaining flies at 25 °C, we crossed the UAS-Flag(SETX)-wt and UAS-Flag(SETX)-L389S with a range of tissue specific and developmentally staged Gal4 drivers. Lines used in this study were obtained from the Bloomington Stock Center or as described previously (Joiner, UCSD). The lines tested were as follows: vGlut-GAL4/Cyo; Cha-Gal4; D42-Gal4; tim-Gal4; Elav-Gal4; Pdf-Gal4; tsh-Gal4; 24B-Gal4; Gad-Gal4/Cyo; Ppk-Gal4; OK371-Gal4, Tdc-Gal4; Ddc-Gal4; and TH-Gal4.

#### Generation of SETX transgenic flies

We sought to generate SETX Tg-flies with the UAS/Gal4 based system which allows for tissue specific expression. We utilized existing SETX plasmids with N-terminal, flag-tags for expression of the wt and L389S human SETX cDNA sequences. Our Flag-wt-SETX and Flag-L389S-SETX expression constructs, described in detail previously [14], were used as the core for generating fly constructs by cloning the full length open-reading frame into the fly pUAST-attB vector (Drosophila Genomic Resource Center). Purified plasmid DNA for wt-SETX, L389S-SETX, and P-LoopΔ-SETX was submitted to the BestGene, Inc. < www.thebestgene.com > for injection for directed integration into the loci 68A4–attP2 site. The results were that n = 8 founders each for of UAS-Flag(SETX)-wt, and UAS-Flag(SETX)-L389S were generated and were subsequently outcrossed into the w1118 iso31 isogenic background for at least two generations. These were bred onto the Bristle stubble inversion balancer (Tm6b) to prevent recombination. As our analysis found that there were no detectible differences (data not shown), we now maintain just single lines of the wt-and L389S-Tg flies. The (GR)1000 lines with nSYB-GAL4 controlled pan-neuronal expression were generated as previously described [50].

#### Ex vivo immunohistochemistry

Drosophila salivary gland dissections were performed as described previously [50]. Larval salivary glands were dissected in PBS and fixed for 7 min in 3.7% formaldehyde in PBS. GR50 was labelled with anti-GFP (1:1000, rabbit, abcam, ab290, preabsorbed against *Drosophila* embryos, RRID: AB_303395). SETX-Flag was labeled with anti-Flag (mAb 1:1000, Sigma). Secondary antibodies were anti-Rabbit IgG (H + L) Alexa Fluor 488 (1:200, RRID: AB-2338046, goat) and anti-mouse IgG (H + L) Cy3 (1:200, RRID: AB-2338685, goat). Tissues were mounted in Vectashield Hardset mounting medium (RRID: AB-2336787). Imaging was performed using a Leica DM6000 B Microscope (low magnification) or a Leica TCS SP8 Microscope (high magnification); and using a Hamamatsu ORCA-R2 C10600-10B-H camera.

#### NMJ analysis

Third instar wandering larvae were dissected, fixed, antibody stained, imaged and analyzed as described previously [49, 50]. All NMJ analysis was performed double-blind. Primary antibodies used were Cy3-Conjugated anti-HRP (Goat, 1:200, Jackson ImmunoResearch Labs Cat# 123-165-021, RRID:AB_2338959) and, anti-synaptotagmin (Rabbit, 1:2000, Syt-91, RRID:AB_271399). Secondary antibodies used were anti-Rabbit IgG (H + L) Alexa Fluor 488 (1:1000, RRID:AB_2576217, goat). NMJs were imaged for quantification using an EVOS M5000 microscope. NMJ images shown were imaged using a Leica SP5 confocal microscope. NMJ bouton number and muscle surface area was quantified manually using images in ImageJ. Bouton number and length were normalized to muscle surface area. NMJ lengths were measured from stacked NMJ images using the NeuronJ plugin for ImageJ as described previously [49, 50].

#### Motor climbing assay

Flies were placed individually, without anesthetization, inside glass boiling tubes mounted on a white background. After acclimatization, the flies were banged down to the bottom of the tubes to elicit the startle-induced negative geotaxis escape behavior. Videos were recorded to determine the distance in millimeters travelled in 20 s.

### Studies of iPSC motor neurons

Fibroblasts were generated from dermal biopsies from ALS4 patients, upon informed consent and in compliance with IRB protocols. Fibroblasts were cultured in Dulbecco's modified Eagle's medium (DMEM) with 10% FBS and 5% penicillin and streptomycin. Low passage fibroblast cultures were reprogrammed into induced pluripotent stem cells (iPSCs) and fully characterized, as done previously in our lab [47]. Isogenic SETX corrected WT and SETX KO lines were generated from an ALS4 SETX L389S line by Applied StemCell and fully characterized for clonality and chromosome integrity. Induced pluripotent stem cells (iPSCs) were maintained in mTeSR (STEMCELL Technologies, 100-0276) and were cultured at 37 °C and 5% CO2. On day 0, 1 × 106 iPSCs were seeded in NB media (DMEM/F12 with 15 mM HEPES, 200 μM Ascorbic Acid, 1X GlutaMAX, 1 N2, and 1X B27 Plus) supplemented with 1 μM Dorsomorphin, 10 μM SB431542, 3 μM CHIR99021, and 10 μM Y-27632 in one well of a 6 well plate coated with Matrigel. Media minus Y-27632 was changed daily until day 6. On day 6, cells were split 1:10 and plated in NB media supplemented with 1 μM Dorsomorphin, 10 μM SB431542, 1.5 μM Retinoic Acid, and 200 nM Smoothened Agonist, and media were changed daily until day 18, at which point cells were dissociated and re-plated on poly-orninthine/laminin coated 10 cm^2^ plates at 6–12 million cells/plate in NB media supplemented with 1.5 μM Retinoic Acid, 200 nM Smoothened Agonist, and 8 ng/ml each BDNF, GDNF, and CNTF. On day 24, immature MNs were re-plated for a final time in NB media supplemented with neurotrophic factors and 2 μM DAPT at a density of 250–300 × 103 cells per cm^2^ on poly-ornithine/laminin coated plates. On day 26, media was changed to MN maintenance media (NB with neurotrophic factors) to wash out DAPT. Half-changes of media were performed every 2 days until mature MNs were analyzed, day 31 or older.

iPSC-derived motor neurons (iPSC-MNs) were plated onto a 96 well plate with a polymer bottom black frame (Cellvis, P96-1.5P) and transduced with lentivirus encoding mCherry-NCL and GFP-GR30 for 100 h. iPSC-MNs were maintained at 37 °C with 5% CO_2_ during FRAP analysis. FRAP was performed using a Nikon A1R confocal microscope. FRAP conditions were empirically established such that > 85% of the mCherry-NCL signal was ablated, and neurons remained viable. After five initial scans, photobleaching was performed with the 561 nm laser at 31% power for 6 iterations. During the recovery phase, we imaged every 4 s for 100 cycles. The percentage of fluorescent recovery per minute was calculated for n ≥ 9 iPSC MNs per biological replicate (n = 3), averaged over three experiments using NIS-Elements AR analysis software.

### Statistical analysis

All data were prepared for analysis with standard spread sheet software (Microsoft Excel). Statistical analysis was done using Microsoft Excel, GraphPad Prism 9.0, the VassarStats website (http://faculty.vassar.edu/lowry/VassarStats.html), or One-Way ANOVA calculator website (https://astatsa.com/OneWay_Anova_with_TukeyHSD/). For ANOVA, if statistical significance was achieved (*P* < 0.05), we performed post hoc analysis to account for multiple comparisons. All t-tests were two-tailed. The level of significance (alpha) was always set at 0.05.

## Results

### SETX is a modifier of *C9orf72* G4C2 repeat expansion toxicity and arginine-containing dipeptide repeat toxicity in vitro

**Fig. 1:**
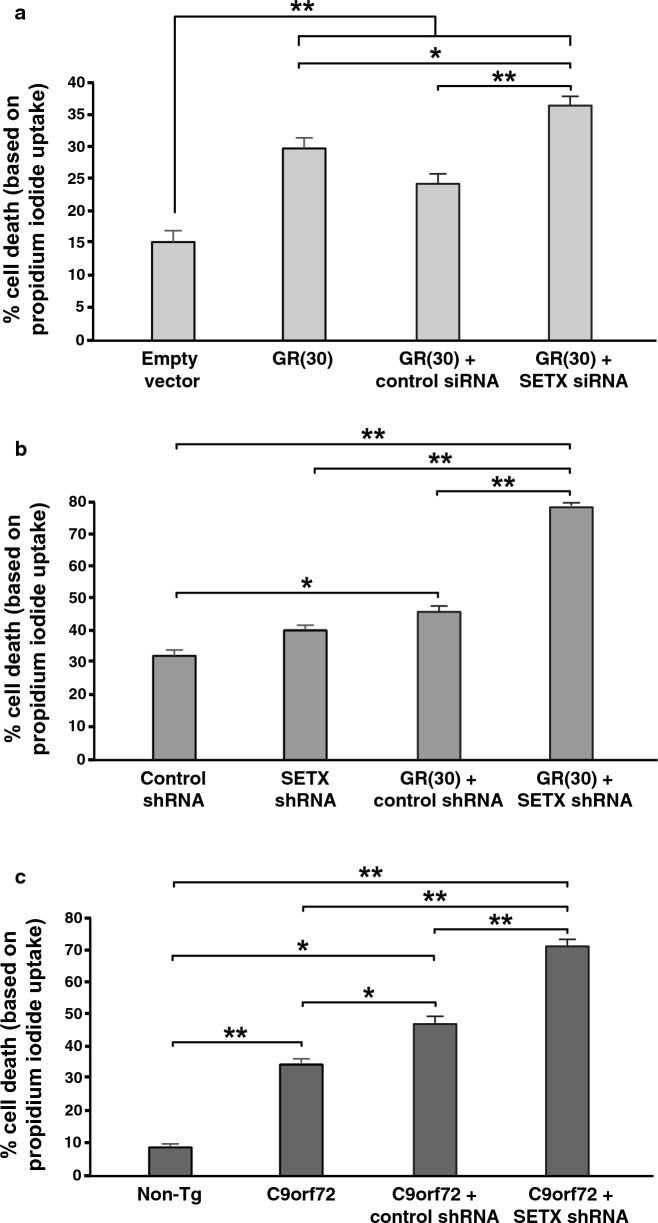
SETX loss-of-function exacerbates C9orf72 neurotoxicity pathways. **a** We transduced HEK293 cells with a lentivirus vector containing an interrupted synthetic construct encoding a GR30 dipepetide, and also treated with a scrambled control siRNA or SETX siRNA, as indicated. After 48 h, we measured cell death by performing a propidium iodide exclusion assay (n = 4 biological replicates). HEK293 cells transduced with lentivirus containing an empty vector served as the negative control. ANOVA with post-hoc Tukey test; *P < 0.05, **P < 0.01. **b** On DIV14, we transduced primary cortical neurons with a lentivirus vector containing an interrupted synthetic construct encoding a GR30 dipepetide, and with a lentivirus vector containing either a scrambled control shRNA or SETX shRNA, as indicated. After 24 h, we measured cell death by performing a propidium iodide exclusion assay (n = 4 biological replicates). Cortical neurons transduced with lentivirus containing the scrambled control siRNA served as the negative control. ANOVA with post-hoc Tukey test; *P < 0.05, **P < 0.01. **c** We cultured primary cortical neurons from C9^450C^ BAC transgenic mice, and on DIV14, we transduced these primary cortical neurons with a lentivirus vector containing either a scrambled control shRNA or SETX shRNA, as indicated. After 24 h, we measured cell death by performing a propidium iodide exclusion assay (n = 3 biological replicates). Cortical neurons from non-transgenic littermate control mice served as the negative control. ANOVA with post-hoc Tukey test; *P < 0.05, **P < 0.01. See Additional file 1: Table S1 for all *P* values. Error bars = s.e.m

It has been previously reported that DPRs produced by RAN translation of the expanded *C9orf72* G4C2 repeat can induce toxicity in HEK293 cells [45]. To determine if SETX is a modifier of DPR toxicity, we obtained interrupted synthetic DPR constructs to avoid the potential contribution of *C9orf72* G4C2 repeat expansion RNA toxicity [45], and evaluated constructs encoding either a GA(30) DPR or GR(30) DPR. While transduction of HEK293 cells with a GA(30) lentivirus vector did not elicit toxicity in HEK293 cells (Additional file 1: Fig. S1a), we observed significantly increased cell death in HEK293 cells transduced with a GR(30) lentivirus vector (Fig. 1a), underscoring the enhanced toxicity of arginine-containing DPRs. When we concomitantly performed siRNA knock-down of SETX in GR(30) expressing HEK293 cells, we noted a marked increase in cell death, but not in GR(30) expressing HEK293 cells transduced with a scrambled siRNA control construct (Fig. 1a). To assess this SETX modifier effect in a more physiologically relevant system, we evaluated the effect of SETX dosage reduction in primary cortical neurons transduced with a GR(30) lentivirus vector, and after confirming that SETX shRNA knock-down in primary neurons achieved a reduction in SETX expression at the RNA and protein level of 50% to 60% (Additional file 1: Fig. S1b, c), we documented a doubling of cell death in GR(30) expressing cortical neurons subjected to SETX shRNA treatment (Fig. 1b). We again failed to detect toxicity upon GA(30) expression, as transduction of primary cortical neurons with a GA(30) lentivirus vector did not result in more neuron cell death (Additional file 1: Fig. S1d). To further establish that SETX is a modifier of neurotoxicity resulting from the *C9orf72* G4C2 repeat expansion mutation, we cultured primary cortical neurons from C9^450^ BAC transgenic mice [22], and found that primary cortical neurons from C9^450^ BAC transgenic mice display dramatically elevated cell death at fourfold that of primary cortical neurons derived from wild-type littermate control mice (Fig. 1c). While treatment of primary cortical neurons from C9^450^ BAC transgenic mice with a control scrambled shRNA did result in a modest, but significant increase in neuron cell death, SETX shRNA treatment of C9^450^ BAC transgenic primary neurons yielded markedly elevated neuron cell death, significantly more than control shRNA treatment, and greater than twice the cell death in untreated primary cortical neurons from C9^450^ BAC transgenic mice (Fig. 1c). These results indicate that decreased SETX expression is sufficient to markedly enhance GR(30) and *C9orf72* G4C2 repeat expansion neurotoxicity.

### Expression of normal or mutant human SETX protein in *Drosophila* does not elicit degenerative disease phenotypes

To further assess the role of SETX in motor neuron disease and neurodegeneration, we examined *Drosophila* expressing transgenic human SETX under the control of the UAS-Gal4 system. To do so, we derived *UAS-FLAG-SETX(wt)* and *UAS-FLAG-SETX(L389S*) transgenic vectors, and confirmed that these vectors drive expression of full-length human SETX protein (Additional file 1: Fig. S2a). The SETX L389S mutation is the most common genetic cause of autosomal dominant ALS4 [6]. After producing transgenic flies carrying either *UAS-FLAG-SETX(wt)* or *UAS-FLAG-SETX(L389S)*, we crossed these flies with a variety of GAL4 driver lines (*vglut*, *cha*, *D42*, *ppk*, *GAD*, *elav*, and *24B*) to achieve expression in different neuronal lineages, glia, and muscle, and evaluated the resultant flies, but we did not detect any abnormalities or disease phenotypes. We also crossed the *UAS-FLAG-SETX(wt)* and *UAS-FLAG-SETX(L389S)* fly lines with *GMR-GAL4* (GMR-GAL4/Cyo) to direct expression to the fly eye, and again did not observe any abnormalities. The only detectable phenotype occurred when we crossed the *UAS-FLAG-SETX(wt)* and *UAS-FLAG-SETX(L389S)* fly lines with the *tsh-GAL4* driver, which promotes expression during development throughout the ventral nerve cord (and other tissues), including in motor neurons [39], as these crosses yielded a phenotype of larval lethality for both normal SETX and L389S SETX.

### SETX is a potent suppressor of *C9orf72 *G4C2 and dipeptide repeat disease phenotypes in *Drosophila*

**Fig. 2:**
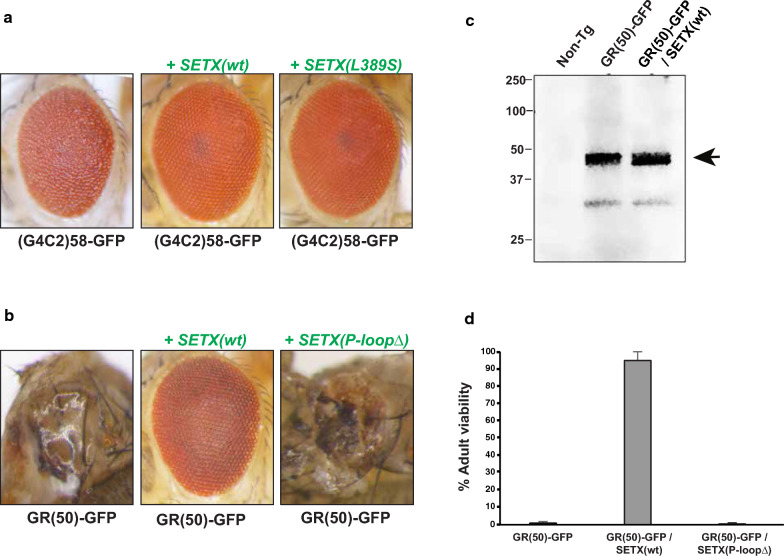
SETX co-expression rescues C9orf72 and dipeptide repeat toxicity in *Drosophila*. **a** Stereomicroscopy images of representative *Drosophila* eyes expressing (G4C2)_58_ driven by *GMR-GAL4* reveals a rough eye phenotype (left), which is suppressed in flies concomitantly expressing normal human SETX (middle) or mutant L389S SETX (right). **b** Stereomicroscopy images of representative *Drosophila* eyes expressing protein-only GR(50) dipepetides driven by *GMR-GAL4* reveals extensive eye degeneration (left), which is fully suppressed in flies concomitantly expressing normal human SETX (center), but not in flies expressing the P-loopΔ SETX mutant (right), which lacks a functional helicase catalytic domain. **c** We performed immunoblot analysis on protein extracts prepared from late pupal stage heads from flies of the indicated genotypes with an anti-GFP antibody. Note comparable levels of GR(50) dipeptide expression in GR(50) only flies and GR(50) / SETX(wt) flies. **d** We monitored survival to the adult stage for different lines of transgenic flies of the indicated genotypes, and documented nearly complete rescue of lethality in GR(50) expressing flies crossed with SETX(wt) expressing flies. Survival to the adult stage no longer occurred in GR(50) expressing flies crossed with SETX helicase domain mutation (P-loopΔ) expressing flies. Error bars = s.d

SETX has been shown to physically interact with components of the nuclear exosome complex, and may be involved in regulating RNA degradation, especially upon transcription termination [42]. As expression of the *C9orf72* G4C2 repeat induces length and dosage dependent degeneration in *Drosophila*, and these degenerative phenotypes are enhanced by loss-of-function of genes encoding nuclear exosome complex components [18], we wondered if expression of human SETX might be capable of suppressing *C9orf72* G4C2 repeat expansion toxicity in the fly eye. To test this hypothesis, we derived fly lines carrying a genomic integration of a UAS-driven (G4C2)_58_ repeat, co-expressed with *UAS-FLAG-SETX(wt)* or *UAS-FLAG-SETX(L389S)* under control of the *GMR-GAL4* driver. We observed complete suppression of the (G4C2)_58_-induced rough eye phenotype in flies concomitantly expressing either normal SETX or L389S SETX (Fig. 2a). To determine if SETX is similarly capable of suppressing degenerative phenotypes induced by expression of a synthetic arginine-containing DPR, we co-expressed *UAS-GR(50)-GFP* [18] and *UAS-FLAG-SETX(wt)* under control of the *GMR-GAL4* driver. We noted total suppression of a severe degenerative eye phenotype in GR(50) flies co-expressing human SETX (Fig. 2b). To exclude an effect of SETX on transgenic expression of GR(50), we performed immunoblot analysis and confirmed comparable expression of GR(50) dipeptide in GR(50)-GFP / SETX(wt) flies (Fig. 2c).

To determine if SETX helicase function is required for suppression of GR(50) toxicity, we generated a transgenic fly line carrying a SETX helicase domain mutation due to a small deletion of the P-loop sequence. When we characterized the *UAS-FLAG-SETX(P-loop*Δ*)* fly line by immunoblot analysis, we confirmed strong expression of full-length SETX protein (Additional file 1: Fig. S2b). When we derived fly lines containing *UAS-GR(50)-GFP*, *UAS-FLAG-SETX(P-loop*Δ*)*, and *GMR-GAL4*, we found that GR(50) flies co-expressing human SETX with the P-loop deletion exhibited severe degenerative eye phenotypes (Fig. 2b). Another degenerative phenotype noted when *UAS-GR(50)-GFP* flies are crossed with the *GMR-GAL4* driver line is dramatic lethality, with near complete lethality at the pupal stage of development (< 4% of individuals reaching the adult stage). When we derived fly lines containing *UAS-GR(50)-GFP*, *UAS-FLAG-SETX(wt)*, and *GMR-GAL4*, we observed nearly complete rescue of the lethality phenotype, but for fly lines containing *UAS-GR(50)-GFP*, *UAS-FLAG-SETX(P-loop*Δ*)*, and *GMR-GAL4*, were unable to rescue the pupal lethality phenotype (Fig. 2d). These findings indicate that a functional helicase domain is required for SETX rescue of GR(50) phenotypes.

**Fig. 3:**
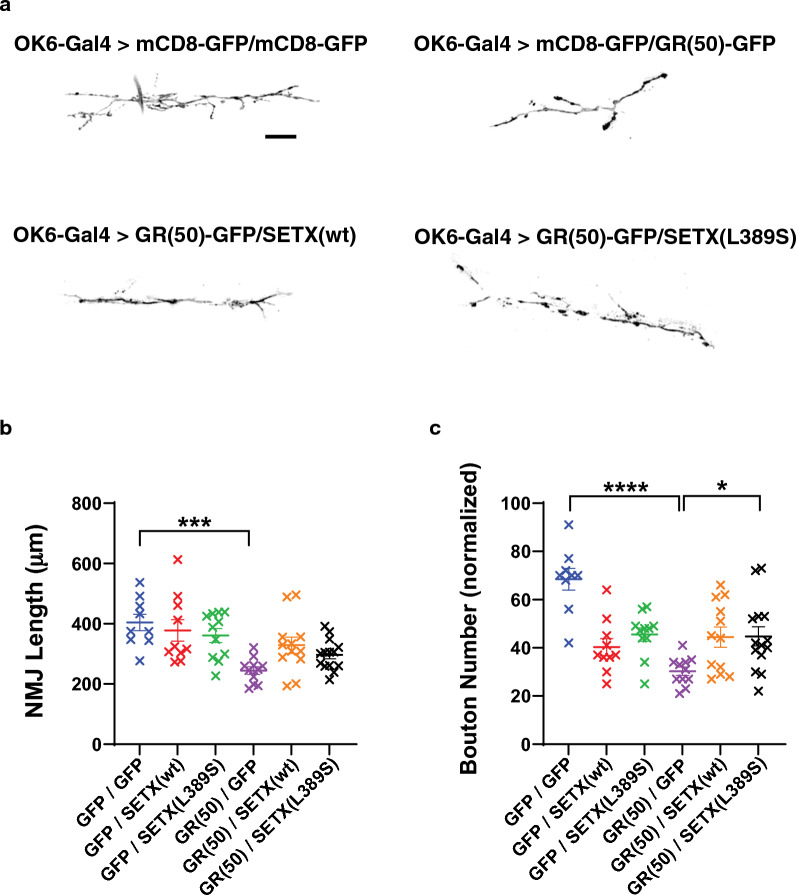
SETX co-expression suppresses neuromuscular junction degeneration in a *Drosophila* model of GR(50) dipeptide toxicit. **a** We directed expression of GR(50)-GFP or membrane targeted mCD8-GFP, alone or in combination with normal SETX(wt) or mutant SETX(L389S), to motor neurons by crossing transgenic flies with flies expressing the *OK6-GAL4* driver. Here we see representative images of the neuromuscular junction (NMJ) of muscle 6/7 at hemi-segment A3 for 3rd instar wandering larvae. Note the reduced length of the NMJ for fly larvae expressing GR(50)-GFP. Scale bar = . **b** We measured the NMJ length at muscle 6/7 at hemi-segment A3 as shown in ‘a’ for fly larvae of the indicated genotypes (n = 9–13). NMJ length was normalized to the muscle surface area. ANOVA with post-hoc Tukey test; *P < 0.05, ****P < 0.0001. **c** We counted the number of synaptic boutons from NMJs as shown in (A) for fly larvae of the indicated genotypes (n = 9–13). Bouton number was normalized to the muscle surface area. ANOVA with post-hoc Tukey test; *P < 0.05, ****P < 0.0001. See Additional file 1: Table S1 for all *P* values. Error bars = s.e.m

To explore the effect of SETX expression on GR(50) disease phenotypes relevant to ALS motor neuron degeneration, we derived fly lines containing *UAS-GR(50)-GFP* and *OK6-GAL4*, which drives expression in motor neurons. When we visualized the neuromuscular junction (NMJ) in fly larvae expressing GR(50), we observed a dramatic reduction in synaptic bouton numbers and in NMJ length (Fig. 3). To evaluate the effect of SETX co-expression on these NMJ degenerative phenotypes, we also derived fly lines containing *UAS-GR(50)-GFP*, *UAS-FLAG-SETX(wt)* or *UAS-FLAG-SETX(L389S)*, and *OK6-GAL4*. NMJ staining of GR(50) fly larvae co-expressing either SETX(wt) or SETX(L389S) revealed increases in synaptic bouton numbers and NMJ length (Fig. 3), indicating that human SETX can also suppress motor neuron disease phenotypes in GR(50)-expressing flies.

### SETX suppresses GR(50) toxicity in *Drosophila* and prevents accumulation of arginine-containing dipeptides in the nucleolus

**Fig. 4:**
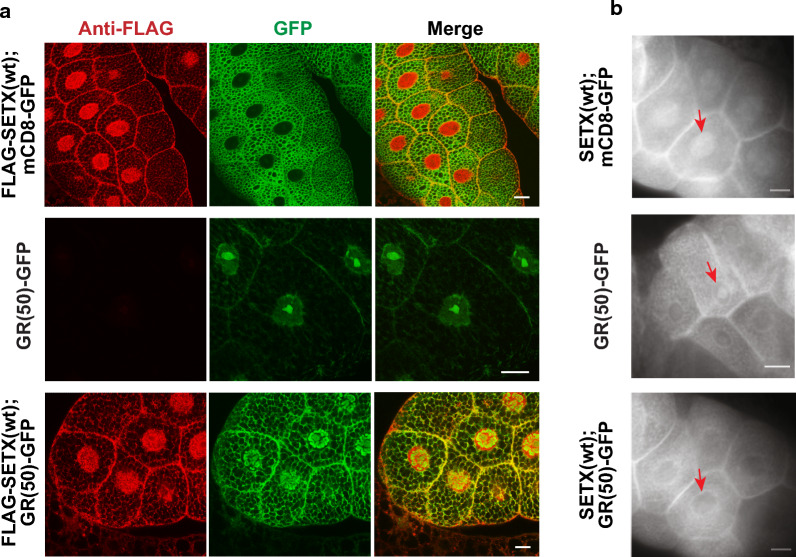
SETX prevents GR(50) dipeptide from localizing exclusively to the nucleolus in the fly salivary gland. **a** We directed expression of GR(50)-GFP or membrane targeted mCD8-GFP, alone or in combination with normal FLAG-SETX(wt), to the salivary gland by crossing transgenic flies with flies expressing the *OK6-GAL4* driver. Flies expressing SETX(wt) with mCD8-GFP exhibit localization of SETX throughout the nucleus (TOP), while flies expressing GR(50)-GFP display dense, punctate accumulation of GR(50) dipeptides in the nucleolus (MIDDLE). Co-expression of SETX(wt) and GR(50)-GFP yields localization of GR(50) dipeptides throughout the nucleoplasm, with partial SETX co-localization (BOTTOM). Though representative images are shown here, these identical findings were observed in all 50 cells examined per genotype. Scale bars = 25 µM. **b** We directed expression of GR(50)-GFP or membrane targeted mCD8-GFP, alone or in combination with normal SETX(wt), to the salivary gland by crossing transgenic flies with flies expressing the *OK6-GAL4* driver. As shown here with monochrome imaging, flies expressing FLAG-SETX(wt) with mCD8-GFP exhibit localization of SETX throughout the nucleus (TOP), while flies expressing GR(50)-GFP display accumulation of GR(50) dipeptides in the nucleolus (MIDDLE). Co-expression of SETX(wt) and GR(50)-GFP reduces the accumulation of GR(50) dipeptides in the nucleolus and nucleus (BOTTOM). Scale bars = 50 µM

What is the mechanism by which expression of human SETX protein so potently suppresses GR(50) toxicity in *Drosophila*? To address this question, we considered an effect of SETX on the subcellular localization of this arginine-containing DPR, as previous studies have documented that GR and PR repeats of this size are typically directed to the nucleolus or other liquid–liquid phase separation (LLPS) derived organelles [20, 24], and because prior work has demonstrated that SETX localizes to the nucleolus [1, 15]. To examine the subcellular localization of GR(50) and SETX in the *Drosophila* model, we crossed transgenic GR(50) and SETX flies with an *OK6-GAL4* fly line, since the OK6 driver directs expression to the developing salivary gland [41]. We derived three distinct fly lineages of the following genotypes: 1) *OK6-Gal4/UAS-mCD8-GFP;UAS-Flag-SETX(wt)/* + ; 2) *OK6-Gal4/UAS-mCD8-mCherry;UAS-GR(50)—GFP/* + ; and 3) *OK6-Gal4/* + *;UAS-GR(50)-GFP/UAS-FLAG-SETX(wt)*. Transgenic flies expressing normal SETX displayed characteristic localization of SETX throughout the nucleoplasm (Fig. 4a). Co-expression of mCD8-GFP, which shows a distinct membrane-targeted localization, acted as a titration control for the OK6-GAL4 driver [11]. Transgenic flies expressing GR(50) exhibited dense intra-nuclear punctate staining, consistent with localization to the nucleolus; however, bigenic flies co-expressing SETX and GR(50) yielded a distinctly different distribution of GR(50), localizing throughout the nucleus, and thus no longer restricted to single, dense subnuclear puncta (Fig. 4a). We repeated the localization experiments in different fly progeny using monochrome imaging, and we observed similar results, noting that bigenic flies co-expressing SETX and GR(50) no longer displayed a dense intranuclear accumulation of GR(50) dipeptides, as was the case for singly transgenic GR(50) individuals (Fig. 4b). These findings suggest that SETX rescue of arginine dipeptide toxicity in the fly may result from its ability to prevent GR(50) accumulation in the nucleolus.

### Normal SETX rescues GR(1000) motor phenotypes in *Drosophila,* but SETX-L389S does not

**Fig. 5:**
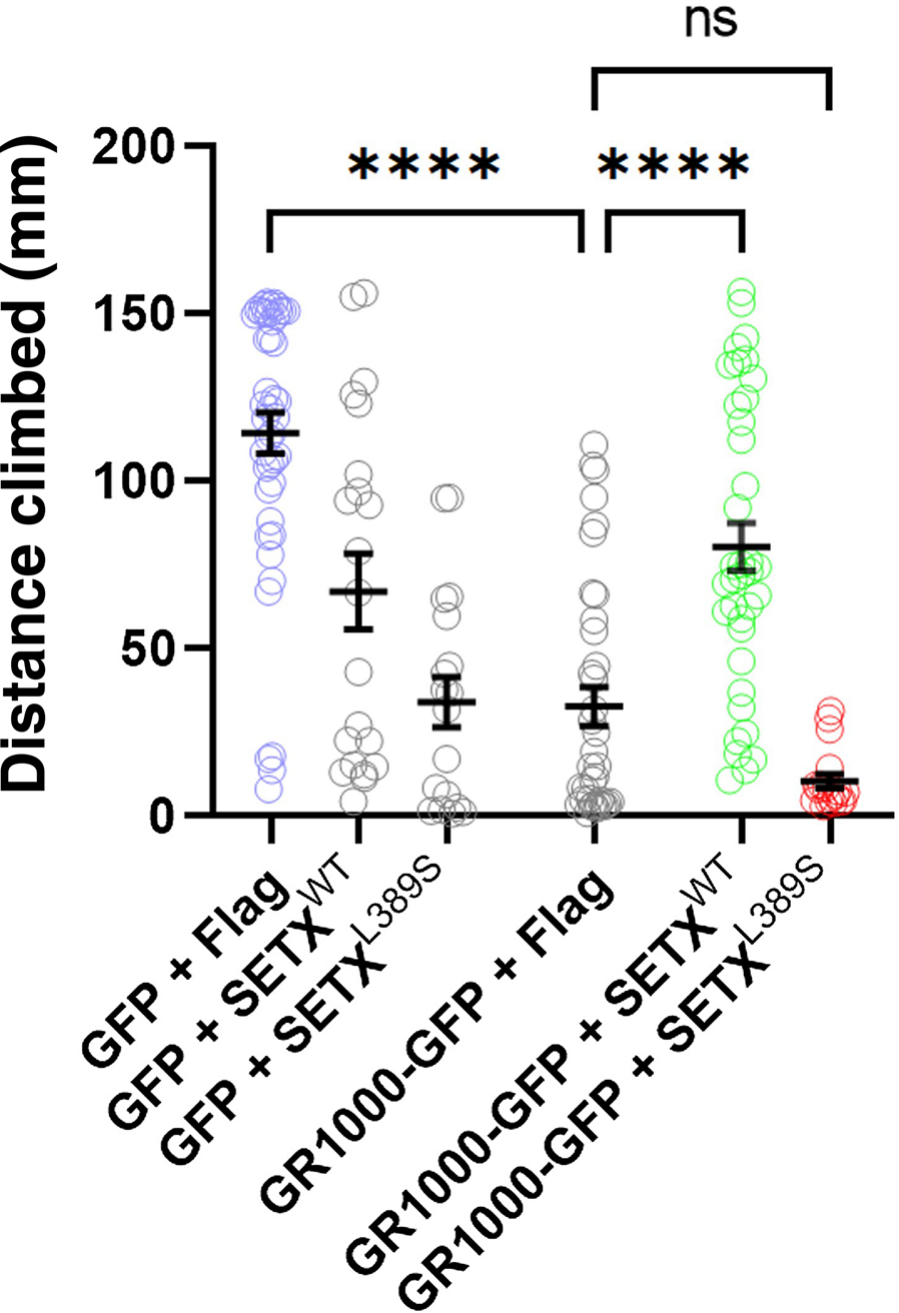
Normal human SETX can rescue motor impairment caused by neuronal expression of GR(1000) in transgenic flies. We directed pan-neuronal expression of GR(1000)-GFP or GFP only, in combination with either normal FLAG-SETX(wt) or FLAG-SETX(L389S) or FLAG only, by crossing transgenic flies with flies expressing the *nSYB-GAL4* driver. At 14 days of age, we collected 30–50 male flies, and performed a negative geotaxis climbing assay to assess motor function by measuring the distance traveled upward (mm) in 20 s. Flies expressing the GR(1000)-GFP transgene in combination with a separate FLAG transgene displayed a reduced mean climbing distance of 32.8 mm, which was a dramatic decrease from the 114.4 mm observed in flies expressing the GFP transgene and FLAG transgene (ANOVA with post-hoc Tukey test; ****P < 0.0001). Neuronal co-expression of GR(1000)-GFP with FLAG-SETX(wt) yielded an increase in mean climbing distance to 80.4 mm, which represented a significant rescue (ANOVA with post-hoc Tukey test; ****P < 0.0001); however, neuronal co-expression of GR(1000)-GFP with FLAG-SETX(L389S) resulted in a mean climbing distance similar to the motor performance observed in flies expressing the GR(1000)-GFP transgene in combination the FLAG-only transgene (ANOVA with post-hoc Tukey test; *P* = n.s.). See Additional file 1: Table S1 for all *P* values. Error bars = s.e.m

Greater attention is now being paid to longer C9orf72 DPRs in ALS. Rare human pedigrees indicate that unaffected individuals with expansions of ~ 70 G4C2 repeats should be considered premutation carriers, with a strong likelihood to expand to the 1000’s and confer disease in subsequent generations [51]. The exact length of toxic C9orf72 DPRs in human ALS patients remains unknown, but it is possible that longer repeats are more biologically relevant. In *Drosophila* models*,* it is also evident that DPR pathological properties alter with length, suggesting that shorter repeats may not recapitulate the entire disease process [34, 50]. Therefore, we obtained a *Drosophila* model where a GR(1000) transgene placed under the expression of the pan-neuronal driver *nSYB-GAL4* develops significant motor phenotypes [50], and we derived fly lines containing: *UAS-GR(1000)-GFP*, *UAS-FLAG* or *UAS-FLAG-SETX(wt)* or *UAS-FLAG-SETX(L389S)*, and *nSYB-GAL4*. To evaluate motor performance in 14 day-old flies, we employed a negative geotaxis assay and quantified distance climbed in 20 s. Although expression of the human SETX transgene resulted in modest, but not statistically significant reductions in climbing distance, we noted that flies co-expressing normal, wild-type human SETX and GR(1000) displayed a dramatic improvement in climbing distance compared to flies co-expressing FLAG and GR(1000) (Fig. 5). Co-expression of SETX-L389S, however, did not rescue the climbing impairment present in GR(1000)-expressing flies (Fig. 5), indicating that only fully intact human SETX can prevent motor deficits in a *Drosophila* model of C9orf72 DPR toxicity featuring a greatly lengthened glycine-arginine dipeptide.

### SETX physically interacts with arginine-containing DPRs

**Fig. 6:**
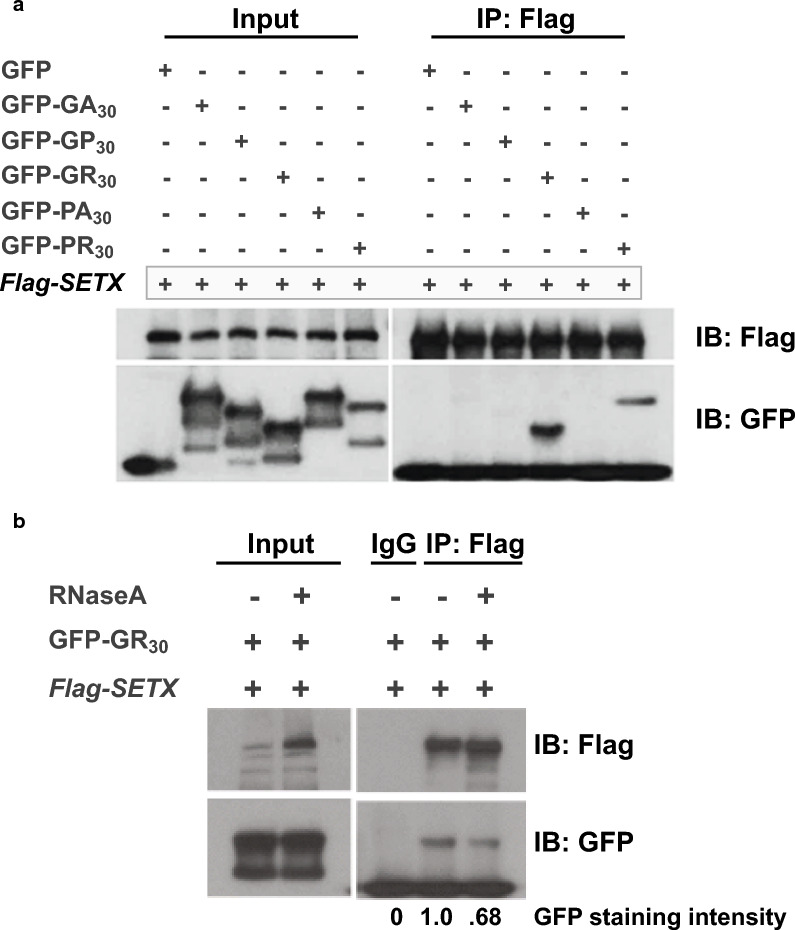
SETX physically interacts with arginine-containing dipeptide repeats, and the SETX interaction with arginine-containing repeats is affected by the presence of RNA. **a** We transfected HEK293 cells with one of five different GFP-linked dipeptide repeat expression constructs or the empty vector negative control, and co-transfected a FLAG-tagged full-length SETX expression construct. We then immunoprecipitated (IP’d) SETX with an anti-FLAG antibody, and performed immunoblot analysis on the IP’d material. We only detected the arginine-containing dipeptide repeats upon co-IP with full-length SETX. **b** To determine if interaction of SETX with arginine-containing dipeptides is RNA-dependent, we repeated the SETX—GR(50) co-IP and treated the IP’d material with or without RNase A. We measured the intensity of the GR(50)-GFP bands by densitometry, and noted a 32% reduction in the SETX—GR(50) interaction upon RNase A treatment. The IgG only IP served as a negative control

As SETX can dictate the localization dynamics of the arginine-containing DPR GR(50) in vivo, we next sought to determine if SETX, a RNA binding protein, engages in a physical interaction with arginine-containing DPRs. To test this hypothesis, we co-expressed each of all five possible dipeptide products of RAN translation of the *C9orf72* G4C2 repeat with full-length FLAG-tagged SETX protein. After confirming expression of full-length SETX protein and each respective 30R dipeptide upon co-transfection, we performed anti-FLAG immunoprecipitation (IP) and then immunoblotted the resultant IP material. We found that SETX formed stable complexes with the arginine-containing dipeptides GR(30) and PR(30), but did not detect an interaction between SETX and the non-arginine containing dipeptides GA(30), GP(30), or PA(30) (Fig. 6a). Current models of the effect of arginine-containing DPRs on membraneless organelles posit that interactions between RNA-binding proteins and arginine-containing DPRs take place in the presence of RNA, which may participate in the stabilization of such RNA–protein assemblies. To determine if SETX interaction with arginine-containing DPRs is facilitated by RNA, we repeated co-immunoprecipitation of SETX and GR(30) in the presence or absence of RNase A. After validating that RNase A treatment of co-immunoprecipitated SETX greatly diminished interaction of SETX with Exosc9 (Additional file 1: Fig. S3), we found that RNase A treatment reduced the SETX—GR(30) interaction by > 30% (Fig. 6b).

### SETX maintains the liquidity of the nucleolus in cells expressing arginine-containing DPRs

**Fig. 7:**
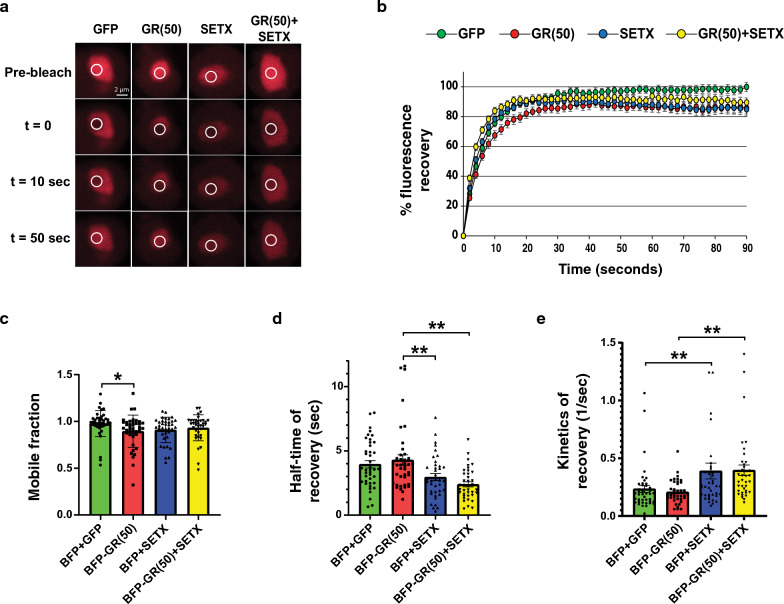
SETX rescues delayed recovery of nucleolus fluorescence in cells expressing arginine-containing dipeptide repeats. **a** We transfected HEK293 cells with a mCherry-nucleolin vector (red), and co-transfected either a BFP-GR(50) expression construct or BFP empty vector, in combination with either a SETX-GFP expression construct or GFP empty vector. After 48 h, we performed fluorescence recovery after photobleaching (FRAP). Here we see representative images depicting the photobleaching and recovery of fluorescence at specific times after the photobleaching the nucleolus of mCherry-nucleolin expressing cells. Scale bar = 2 µM. **b** Results of FRAP shown at two second intervals from photobleaching (t = 0) until 90 s after photobleaching. Note initial delayed fluorescence recovery in GR(50) expressing cells, and enhanced fluorescence recovery in SETX expressing cells. n = 40 cells/condition. **c** Quantification of the mobile fraction of the nucleolus from (B). Note the significant reduction in the mobile fraction of the nucleolus in GR(50) expressing cells. ANOVA with post-hoc Tukey test; *P < 0.05. **d** Measurement of the half-time of fluorescence recovery from (B). Note significantly increased half-time of recovery in GR(50) expressing cells, which is rescued in GR(50) transfected cells co-expressing SETX. ANOVA with post-hoc Tukey test; **P < 0.01. **e** Analysis of the kinetics of recovery from (B). Note significantly slowed recovery in GR(50) expressing cells, which is rescued in GR(50) transfected cells co-expressing SETX, and also how SETX expression accelerates recovery in control cells. ANOVA with post-hoc Tukey test; **P < 0.01. See Additional file 1: Table S1 for all *P* values. Error bars = s.e.m

Arginine-containing DPRs reduce the liquidity of the nucleolus, impairing its ability to modulate protein aggregation under stress conditions [19]. As SETX can reduce the toxicity of the GR(50) DPR by attenuating its accumulation in the nucleolus, we decided to test if SETX is capable of rescuing the aberrant nucleolus phase dynamics caused by the overexpression of arginine-containing DPRs. To evaluate phase dynamics of the nucleolus in the presence of the toxic GR DPR, we employed a construct containing Nucleolin fused to mCherry (NCL-mCherry) and we measured fluorescence recovery after photobleaching (FRAP) of the nucleolus for HEK293 cells co-transfected with NCL-mCherry in combination with a GR(50) vector alone or in combination with SETX (Fig. 7a). After performing FRAP on the nucleolus for 90 s, we noted that recovery of the NCL signal lagged for HEK293 cells expressing GR(50) in comparison to HEK293 cells expressing empty vector, SETX only, or GR(50) and SETX together (Fig. 7b). Indeed, in agreement with a previously published study [19], HEK293 cells expressing GR(50) exhibit a significant reduction of the nucleolus mobile fraction (Fig. 7c). Despite the protective effect of SETX in countering arginine-containing DPR toxicity, co-expression of SETX did not improve the mobile fraction of the nucleolus (Fig. 7c). However, when we examined the kinetics of fluorescence recovery for Nucleolin in HEK293 cells expressing SETX and GR(50) in comparison to HEK293 cells expressing only GR(50), we observed a marked improvement in the half-time of recovery of the NCL signal in the nucleolus upon SETX co-expression (Fig. 7d), and we documented a significantly more rapid kinetics of recovery of the NCL signal in the nucleolus of HEK293 cells expressing SETX and GR(50) (Fig. 7e). These findings indicate that SETX may prevent the toxicity of arginine-containing DPRs, at least in part, by modulating its deleterious effects on the liquidity of the nucleolus.

### Loss or alteration of SETX impairs the regulation of nucleolus liquidity in motor neurons

**Fig. 8:**
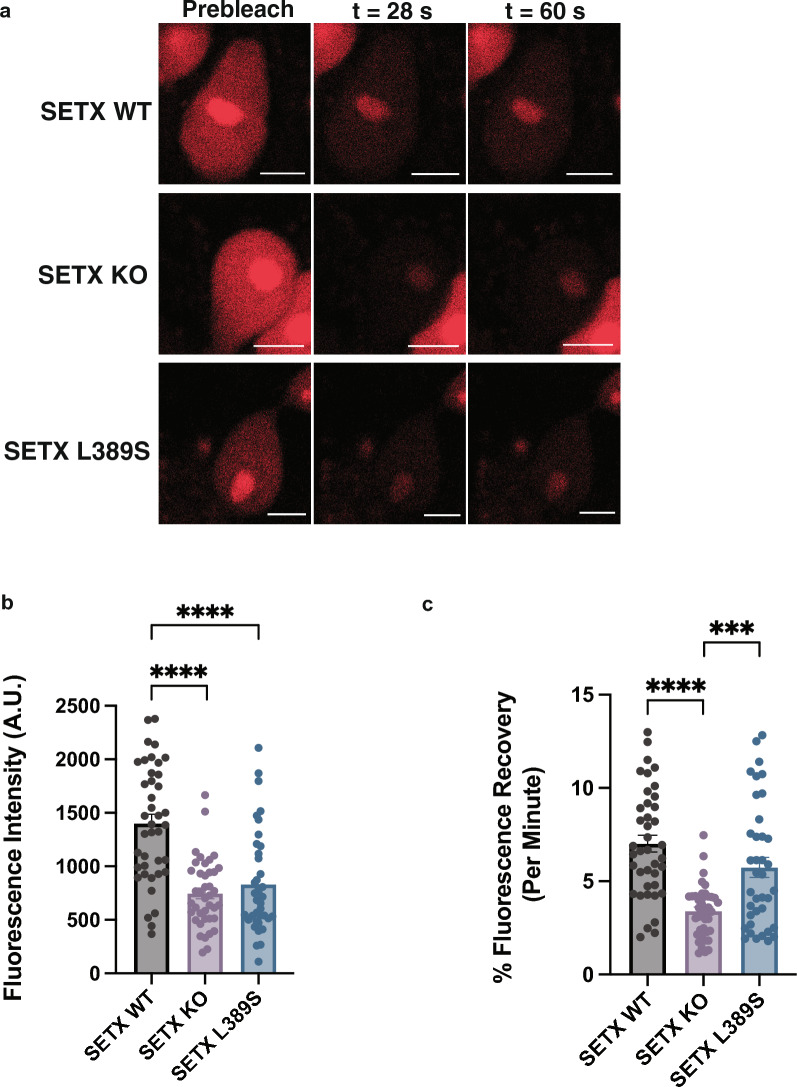
iPSC-derived motor neurons transduced with arginine-rich DPRs exhibit reduced nucleolus liquidity upon mutation or genetic loss of SETX. **a.** Here we see representative images of iPSC-MNs of the indicated genotypes transduced with lentivirus encoding NCL-mCherry and a GR(30) lentivirus vector, shown prior to photobleaching (left) and at two time points post-nucleolus photobleaching. Scale bars = 5 μM. **b** Fluorescence intensity measured over the first 28 s post-nucleolus photobleaching (“initial recovery”). **c** Percent fluorescence recovery per minute following nucleolus photobleaching over the entire period of recovery (400 s). Results are shown for n = 3 biological replicates, for n = 9–13 iPSC motor neurons per biological replicate, yielding analysis of 38–40 motor neurons per genotype. ANOVA with post-hoc Tukey test; ***P < 0.001, ****P < 0.0001. Error bars = s.e.m.

To confirm the relevance of SETX function to nucleolus liquidity in a more physiologically relevant cell type, we obtained an induced pluripotent stem cell (iPSC) line from an ALS4 patient carrying the pathogenic L389S mutation (SETX L389S), and using genome editing, we generated an isogenic corrected iPSC line (SETX WT) and an isogenic knock-out line lacking SETX expression (SETX KO). After deriving motor neurons (MNs) from these three isogenic iPSC lines, we transduced iPSC-MNs with the NCL-mCherry vector and a GFP-tagged GR(30) expression construct, and we performed FRAP in order to evaluate phase dynamics of the nucleolus in the presence of the toxic GR DPR. Fluorescence recovery for bleached nucleolus regions was visibly decreased for both SETX KO iPSC-MNs and SETX L389S iPSC-MNs (Fig. 8a), and this was reflected by a marked decrease in the rate of fluorescence recovery for both SETX KO iPSC-MNs and SETX L389S iPSC-MNs for the initial period of monitoring (Fig. 8b). Upon calculation of the percent fluorescence recovery for the entirety of the experiment however, we documented a significant decrease in the recovery rate for SETX KO iPSC-MNs, but only observed a decrease in the recovery rate for SETX L389S iPSC-MNs, which constituted a strong trend (*P* = 0.089) (Fig. 8c). These findings indicate that normal SETX function is crucial for maintaining the liquidity of the nucleolus in motor neurons, and show that altered SETX function due to the ALS4 L389S mutation may also result in a nucleolus homeostasis defect in motor neurons.

## Discussion

Most forms of familial ALS are clinically and pathologically indistinguishable from sporadic ALS, suggesting that shared pathways of disease pathogenesis likely exist. Identifying genetic modifiers of the most common types of familial ALS may thus yield targets and pathways for therapeutic modulation, and it is possible that such therapies will be effective in patients with these common familial ALS subtypes; but could also be disease-modifying treatments in sporadic ALS. We have been interested in understanding the molecular genetic basis of ALS4, a rare, dominantly inherited juvenile-onset form of ALS resulting from mutations in the senataxin gene [14]. We have found several intriguing features of ALS4 and of its causal RNA-binding protein (RBP) SETX. Although ALS4 is exceedingly rare, in the dozen or so families identified to date, only three different mutations have been discovered, all of which are single amino acid substitutions: T3I, L389S, and R2136H [4]. Of the two highly penetrant mutations, one is located in the amino-terminal protein–protein interaction domain (L389S) and one is located in the carboxy-terminal helicase domain (R2136H). We have shown that transgenic expression of the R2136H mutation and knock-in of the L389S mutation are both sufficient to produce ALS4 disease phenotypes in mice, presenting as slowly progressive motor neuronopathies with the hallmark TDP-43 histopathology seen in sporadic ALS and most forms of familial ALS—TDP-43 nuclear clearing and cytosolic aggregation [3]. Although ALS4 is very rare, unbiased genomic sequencing studies of large cohorts of sporadic ALS patients have revealed that SETX mutations are among the most commonly detected alterations [16]. In one study of 391 sporadic ALS patients, direct sequencing of candidate genes yielded 17 novel potentially pathogenic variants, seven of which were amino acid substitutions in the SETX gene [12]. Importantly, these SETX mutations were absent in > 15,000 controls [12]. Studies in *Drosophila* ALS models (TDP-43^−M337V^; and FUS^−R521C^) have independently identified the SETX fly orthologue as a modifier of degenerative disease phenotypes [23], further underscoring a potential role for SETX in motor neuron health and disease.

Numerous studies of arginine-containing DPRs have yielded a model in which arginine-containing DPRs disrupt liquid–liquid phase separation of proteins, often RBPs containing low-complexity domains that regulate formation and stability of membraneless-organelles. In particular, the nucleolus and its many RNA regulatory functions have arisen as a central target of arginine-containing DPR toxicity in *C9orf72* ALS [9, 19, 25, 27]. Factors controlling compartmentalization of RNA molecules and proteins into membraneless-organelles remain poorly understood; however, certain RBPs with helicase activity, such as the DEAD-box ATPases, can promote the formation and disassembly of membraneless organelles, including the nucleolus [21]. SETX, akin to TDP-43, displays very tight autoregulation in vivo, but unlike other RBPs, it does not contain a low-complexity domain [3]. Here we evaluated SETX as a modifier of disease toxicity in *C9orf72* ALS, which is the most common familial form of this disease. We observed powerful SETX suppression of *C9orf72* G4C2 repeat toxicity and DPR toxicity in two well-established *Drosophila* models. To understand the mechanistic basis for such profound SETX rescue, we considered the normal function of SETX as an RNA–DNA dependent helicase protein. We found that whereas wildtype human SETX protects against GR(50)-driven lethality in Drosophila, mutant human SETX lacking a helicase domain is not similarly protective, thus confirming that SETX helicase function is essential for countering arginine-containing DPR toxicity. Based on this result, we considered a role for SETX in regulating the localization of arginine-containing DPRs in the nucleolus. We found that in the presence of SETX, accumulation of GR(50) outside of this organelle is increased. Furthermore, via mechanisms that almost certainly require helicase activity, normal human SETX was able to rescue motor phenotypes in (GR)1000-expressing flies. Surprisingly, L389S-SETX failed in this regard. Up to this point, we had not seen a L389S-SETX linked phenotype in the fly. We suggest that this rescue failure may represent a partial loss of function, and recognize one limitation of our study is that we did not include the L389S-SETX and P-loopΔ-SETX genotypes in all experiments. However, we documented SETX modification of G4C2, GR short repeat, and GR long repeat phenotypes in diverse, complementary model systems. Indeed, to directly evaluate the effect of SETX on the liquidity of the nucleolus (in an amenable assay system), we performed FRAP on HEK293 cells expressing NCL-mCherry. We found that the recovery rate of this molecule in the nucleolus was enhanced by SETX, regardless of GR(50) expression. To determine if this modulation of nucleolus liquidity is relevant to motor neurons, we examined the mobility of NCL-mCherry in isogenic iPSC-derived motor neurons derived from an ALS4 patient, corrected control, and SETX KO line. Both SETX L389S and SETX KO motor neurons displayed impaired recovery of NCL-mCherry fluorescence in the nucleolus in FRAP experiments.

How does SETX promote improved liquidity of the nucleolus? As a helicase, SETX may regulate the RNA-RNA and RNA–protein interactions involved in the assembly of this membraneless organelle. Previous studies support such a model, as expression of PR(50) in U2OS cells resulted in impaired recruitment of stress granule disassembly-engaged protein regulators, including SETX, to the SG disassembly complex, indicating SETX may function as a stress-granule disassembly-engaged protein [30]. Furthermore, SETX can augment the action of RNA polymerase II in stabilizing the nucleolus and maintaining rRNA expression [1]. Hence, in membraneless organelles, SETX is likely buffering against the effect of arginine-containing DPRs on the molecular interactions that determine the equilibrium of liquid–liquid phase separation. We propose that SETX interacts with RNA clients via its helicase activity to regulate RNA conformation and availability for interactions with other RNAs and proteins involved in the assembly and disassembly process. SETX interaction with its RNA substrates takes place in the presence of arginine-containing DPRs, as we also found evidence for a physical interaction between SETX and arginine-containing DPRs, and we observed that this interaction is facilitated by the presence of RNA, although the SETX amino-terminal protein-interaction domain was not specifically evaluated.

One emerging cellular function of SETX is its role in regulating the immune system, as SETX has been shown to suppress the antiviral immune response [31]. Building on this finding, we found that CD8 T cells of an autoreactive origin are clonally expanded in ALS4 model mice and ALS4 human patients [13]. Furthermore, transplant of bone marrow hematopoietic cells from normal mice into ALS4 SETX L389S knock-in mice could ameliorate motor neuron degeneration and disease phenotypes in recipient ALS4 knock-in mice. While the mechanistic basis for development of autoimmune T cells in ALS4 remains unknown, recent work has implicated the nucleolus in the degradation of inflammatory RNAs [28]. This process requires that NCL recruit cytosine or uracil-rich pre-mRNAs and the exosome complex into the nucleolus, underscoring the importance of NCL mobility and nucleolus liquidity in the attenuation of the inflammatory response. As SETX physically interacts with the nuclear exosome complex [5], alteration of SETX function in ALS4 due to the L389S mutation could impair its ability to maintain NCL mobility and nucleolus liquidity, thereby contributing to the autoimmune dysfunction and rampant T cell activation noted in ALS4 mice. Similarly, non-ALS4 mutations in SETX, that are commonly detected in *C9orf72* ALS patients [12], could be accentuating immune dysfunction due to partial loss of C9orf72 function, as C9orf72 knock-out mice display autoimmune phenotypes [2, 10, 36]. Hence, SETX dysregulation in the context of G4C2 repeat expansion in the *C9orf72* gene in ALS patients may affect immune cells as well as motor neurons, and this should be a focus of future studies. Our results identify SETX as a central regulator of nucleolus homeostasis with the ability to counter the disruptive influence of arginine-containing DPRs in *C9orf72* ALS and suggest that proper SETX function may be essential for maintaining motor neuron health in the face of various genetic, immune, and environmental insults.

### Supplementary Information

**Additional file 1.** Supplementary Figures and Table.
