# Supplementary material for: Senataxin helicase, the causal gene defect in ALS4, is a significant modifier of C9orf72 ALS G4C2 and arginine-containing dipeptide repeat toxicity
Source: Acta Neuropathol Commun. 2023 Oct 17;11:164. doi: 10.1186/s40478-023-01665-z (PMC10580588; doi:10.1186/s40478-023-01665-z)
Supplement: Supplementary file 1 — Additional file 1. Supplementary Figures and Table. [file 40478_2023_1665_MOESM1_ESM.pdf]

Supplementary Figure Legends

Figure 1. GA(30) dipeptide repeats lack toxicity and validation of SETX shRNA knock-down

a. We transduced HEK293 cells with a lentivirus vector containing an interrupted synthetic construct encoding a GA30 dipepetide. After 48 hrs, we measured cell death by performing a propidium iodide exclusion assay (n = 4 biological replicates). HEK293 cells transduced with lentivirus containing an empty vector served as the negative control. *P* = n.s. by two-tailed t-test.

b. We cultured primary cortical neurons from control wild-type, and on DIV14, we transduced these primary cortical neurons with a lentivirus vector containing either a scrambled control shRNA or SETX shRNA, as indicated. After 24 hrs, we isolated RNA from the different primary neuron cultures and performed qRT-PCR analysis of SETX mRNA expression (n = 3 technical replicates). Cortical neurons transduced with lentivirus containing an empty vector served as the negative control. Note reduction of SETX mRNA expression by ~50% in neurons transduced with SETX shRNA.

c. We cultured primary cortical neurons from control wild-type, and on DIV14, we transduced these primary cortical neurons with a lentivirus vector containing either a scrambled control shRNA or SETX shRNA, as indicated. After 24 hrs, we prepared protein lysates from the different primary neuron cultures and performed immunoblot analysis of SETX (n = 3 technical replicates). Cortical neurons transduced with lentivirus containing an empty vector served as the negative control, and results were normalized to cortical neurons transduced with lentivirus containing empty vector. Note reduction of SETX protein expression by ~60% in neurons transduced with SETX shRNA in comparison to neurons transduced with lentivirus containing empty vector, with minimal reduction of SETX protein expression by ~20% in neurons transduced with control shRNA.

d. On DIV14, we transduced primary cortical neurons with a lentivirus vector containing an interrupted synthetic construct encoding a GA30 dipepetide. After 24 hrs, we measured cell death

by performing a propidium iodide exclusion assay (n = 4 biological replicates). Cortical neurons transduced with lentivirus containing an empty vector served as the negative control. P = n.s. by two-tailed t-test. Error bars = s.e.m.

Figure 2. Validation of SETX protein expression from different transgenic vectors and fly lines

a. We transfected S2R+ insect cells with an *Actin-GAL4* vector in combination with either the *UAS-FLAG-SETX(wt)* or *UAS-FLAG-SETX(L389S)* vector, or left the SCR+ insect cells untransfected. We then prepared protein extracts and performed immunoblot analysis of SETX, and confirmed expression of full-length SETX (arrowhead) from both transgenic vectors.

b. We performed immunoblot analysis on protein extracts prepared from late pupal stage heads from flies of the indicated genotypes with an anti-FLAG antibody. Note that transgenic expression of full-length SETX helicase domain mutation (P-loopΔ) is at least equivalent to transgenic expression of full-length SETX(wt) (arrowhead), excluding insufficient expression of the SETX helicase domain mutant as an explanation for its inability to rescue larval lethality in the GR(50) expressing flies. L = ladder.

Figure 3. Validation of RNaseA treatment of SETX co-immunoprecipitation as a method to assess RNA dependence of SETX protein-protein interaction

As a positive control for RNA-dependent SETX protein-protein interaction, we performed co-immunoprecipitation (co-IP) of SETX and exosome subunit 9 (Exosc9), and testing for RNA dependence by treating the IP'd material with or without RNase A. We measured the intensity of the Exosc9 bands by densitometry, and noted a 54% reduction in the SETX – Exosc9 interaction upon RNase A treatment. The IgG only IP served as a negative control.

Supplementary Table 1

ANOVA *P* values (pairwise post-hoc Tukey tests)

Figure 1a (Hek293 cells)

	Vector	GA30	GR30	GR30/siCRL	GR30/siSETX
**Cells**	<0.0001	0.0002	<0.0001	<0.0001	<0.0001
**Vector**		0.9985	<0.0001	0.0025	<0.0001
**GA30**			<0.0001	0.001	<0.0001
**GR30**				0.1184	0.0307
**GR30/siCRL**					0.0001

Figure 1b (PCNs)

	GA30	CRL-shRNA	SETX-shRNA	GR30 /CRL-shRNA	GR30/SETX-shRNA
**Vector**	>0.9999	0.9992	0.356	0.0124	<0.0001
**GA30**		>0.9999	0.3262	0.0152	<0.0001
**CRL-shRNA**			0.2688	0.0112	<0.0001
**SETX-shRNA**				0.724	<0.0001
**GR30 /CRL-shRNA**					<0.0001

Figure 1c (C9orf72 BAC 450x PCNs)

	C9-450x	C9-450x/sh-CRL	C9-450x/sh-SETX
**non-Tg**	<0.0001	<0.0001	<0.0001
**C9-450x**		0.023	<0.0001
**C9-450x/sh-CRL**			<0.0001

Figure 3b (NMJ)

	GFP, SETX^{wt}	GFP, SETX^{L389S}	GFP, GR50	GR50, SETX^{wt}	GR50, SETX^{L389S}
**GFP, GFP**	0.977	0.8173	0.0004	0.2537	0.024
**GFP, SETX^{wt}**		0.9961	0.0031	0.6826	0.1387
**GFP, SETX^{L389S}**			0.0108	0.9192	0.3348
**GFP, GR50**				0.1171	0.5581
**GR50, SETX^{wt}**					0.9448

Figure 3c (Boutons)

	GFP, SETX^{wt}	GFP, SETX^{L389S}	GFP, GR50	GR50, SETX^{wt}	GR50, SETX^{L389S}
GFP, GFP	<0.0001	0.0011	<0.0001	0.0004	0.0003
GFP, SETX^{wt}		0.9184	0.4129	0.9673	0.9494
GFP, SETX^{L389S}			0.0474	>0.9999	>0.9999
GFP, GR50				0.0705	0.0472
GR50, SETX^{wt}					>0.9999

Figure 5 (Dist. Climbed)

	GFP, SETX^{wt}	GFP, SETX^{L389S}	Flag, GR1000	GR1000, SETX^{wt}	GR1000, SETX^{L389S}
GFP, Flag	0.0001	<0.0001	<0.0001	0.0019	<0.0001
GFP, SETX^{wt}		0.0974	0.0205	0.8144	0.0002
GFP, SETX^{L389S}			>0.9999	0.0008	0.4582
Flag, GR1000				<0.0001	0.3589
GR1000, SETX^{wt}					<0.0001

Figure 7 (FRAP - Mobile fraction)

	GR50 + GFP	BFP + SETX	GR50 + SETX
BFP + GFP	0.035	0.172	0.497
GR50 + GFP		0.969	0.699
BFP + SETX			0.921

Figure 7 (FRAP - Half-Life)

	GR50 + GFP	BFP + SETX	GR50 + SETX
BFP + GFP	0.859	0.091	0.0018
GR50 + GFP		0.012	0.0001
BFP + SETX			0.5651

Figure 7 (FRAP - Kinetics)

	GR50 + GFP	BFP + SETX	GR50 + SETX
BFP + GFP	0.969	0.0583	0.0453
GR50 + GFP		0.019	0.0142
BFP + SETX			0.9997

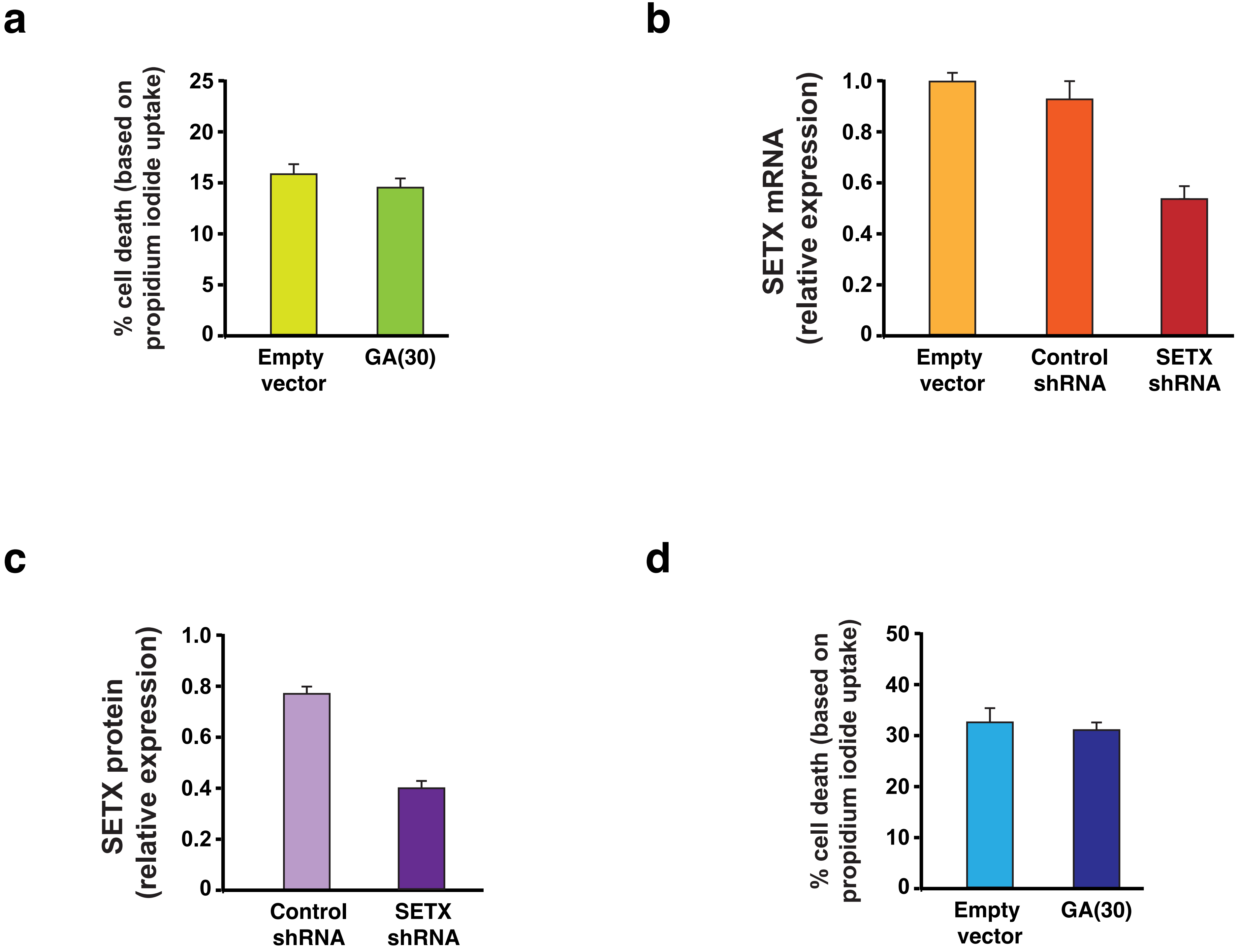
a
% cell death (based on propidium iodide uptake)
25
20
15
10
5
0
Empty vector
GA(30)
b
SETX mRNA (relative expression)
1.0
0.8
0.6
0.4
0.2
0
Empty vector
Control shRNA
SETX shRNA
c
SETX protein (relative expression)
1.0
0.8
0.6
0.4
0.2
0
Control shRNA
SETX shRNA
d
% cell death (based on propidium iodide uptake)
50
40
30
20
10
0
Empty vector
GA(30)

Figure S1

a

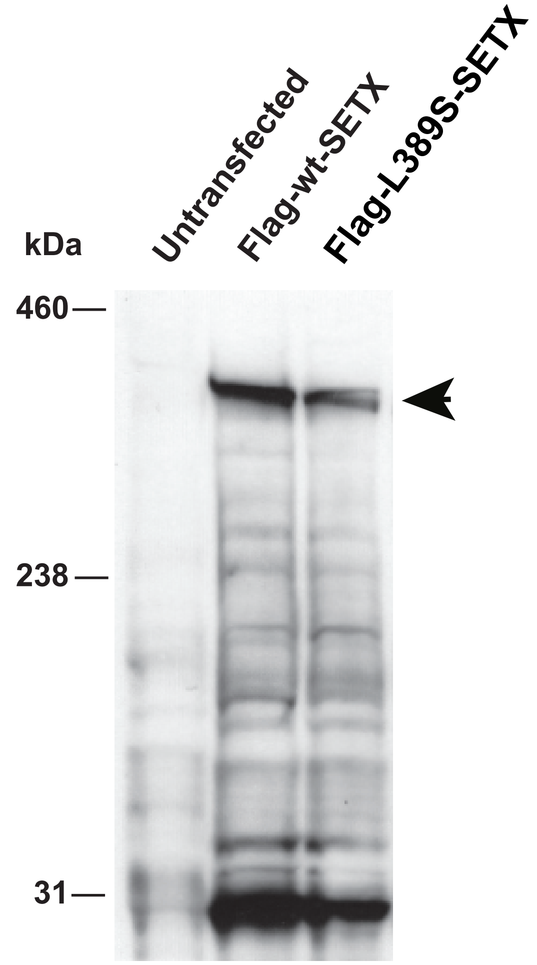
Untransfected
Flag-wt-SETX
Flag-L389S-SETX
kDa
460
238
31

b

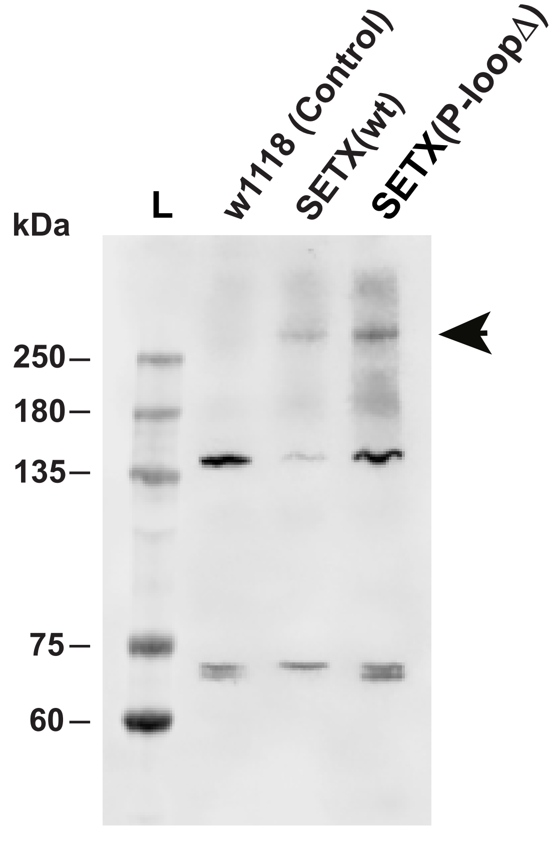
w1118 (Control)
SETX(wt)
SETX(P-loopΔ)
L
kDa
250
180
135
75
60

Figure S2

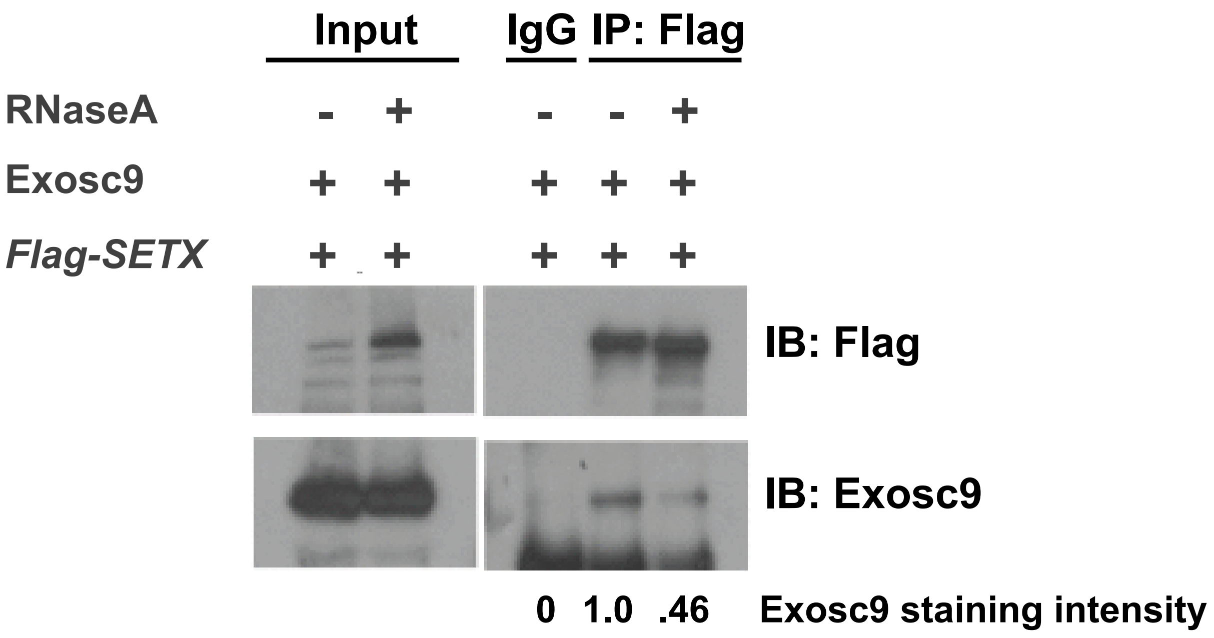
Input
IgG
IP: Flag
RNaseA
-
+
-
-
+
Exosc9
+
+
+
+
+
Flag-SETX
+
+
+
+
+
IB: Flag
IB: Exosc9
0
1.0
.46
Exosc9 staining intensity

Figure S3
